# The Story of the Insane from Year to Year

**Published:** 1901-10-19

**Authors:** 


### THE STORY OF THE INSANE FROM YEAR TO YEAR.

(Thirteenth Series.)

### LANARK DISTRICT ASYLUM AT HARTWOOD.

On the date of this report there were in residence 670 patients. The first admissions numbered 175, the readmissions 132, the recoveries 89, the relieved 41, and the deaths 71. Of these deaths 31 were due to cerebral and spinal diseases, 28 to thoracic diseases, among which phthisis only figures twice. General tuberculosis caused two deaths and enteritis one. We miss the usual table giving the percentage of recoveries on admissions, and the percentage of deaths on the average number resident; but on referring to Dr. Clark's report we find that the former stood at 35·6, and the latter at 11·2 per cent. Table VII. tells us that in 18 of the admissions the insanity was attributed to various moral causes, religion being the highest with 11 cases; that intemperance in drink heads the list of physical agents, and caused the disease in 60 cases. Heredity is credited with 20 cases only, and in the high proportion of 49 the disease was of unknown origin. A very instructive table is given on page 31. It shows the state of physical health of the year's admissions, and we note that only 147 out of the total of 307 could be classified as even in average health, 144 were in weak health, and 16 were "very weak." Dr. Black is convinced of the high value of employment in the treatment of the insane; and he is right, for assuredly no other treatment yet invented equals steady occupation in promoting recovery, and when that is impossible, in "greater quiet and contentment," as Dr. Black puts it. He points to the increased outside activity and the excellent work done on the farm and in the garden, and he contrasts these results with the idleness and the depressing routine exercise, and he adds that it will be his "persistent endeavour to keep up a high percentage of active, out-door employment." So much for the male patients; but a not unexpected difficulty meets him with the women, and he does not think it will be solved until he overcomes the prejudices of his nurses. "How are we to convince them," he asks, "that active, out-door employment with their patients—hoeing, weeding, grass-cutting, thistle-cutting—is something to be proud of, apart from its beneficial effects on the patients." And the echo and ourselves answer "How, indeed?" Dr. Clark does not give up hopes of success, and in the meantime he deserves much praise for ventilating the subject. With a large number of women sewing and knitting are performed mechanically; and the melancholic who is employed at either has ample time to brood over her sorrows, whereas active employment with a hoe would be more likely to dispel her gloom. That is unquestionable. Seven of the nurses and three of the attendants were prepared for the examination of the Medico-Psychological Association, and all obtained the nursing certificate. It is a pity that Dr. Clark does not institute a three years' course and grant his own certificate. A large amount of liberty is given to the patients, and 43 men are on parole. There was no escape during the year. The committee say that the reports of their visiting members "have invariably been highly commendatory" to Dr. Clark's management. The weekly charge to the unions is 8s. 9d.

